# Evidence for Effective Inhibitory Actions on Hyperpolarization-Activated Cation Current Caused by *Ganoderma* Triterpenoids, the Main Active Constitutents of *Ganoderma* Spores

**DOI:** 10.3390/molecules24234256

**Published:** 2019-11-22

**Authors:** Wei-Ting Chang, Zi-Han Gao, Yi-Ching Lo, Sheng-Nan Wu

**Affiliations:** 1 Division of Cardiovascular Medicine, Chi-Mei Medical Center, Tainan 71004, Taiwan; cmcvecho2@gmail.com; 2 Department of Biotechnology, Southern Taiwan University of Science and Technology, Tainan 71004, Taiwan; 3 Institute of Clinical Medicine, College of Medicine, National Cheng Kung University, Tainan 70101, Taiwan; 4 Department of Physiology, National Cheng Kung University Medical College, Tainan 70101, Taiwan; hhelen000111tw@gmail.com; 5 Department of Pharmacology, College of Medicine, Kaohsiung Medical University, Kaohsiung 80708, Taiwan; yichlo@kmu.edu.tw; 6 Institute of Basic Medical Sciences, National Cheng Kung University Medical College, Tainan 70101, Taiwan; 7 Department of Medical Research, China Medical University Hospital, China Medical University, Taichung 40402, Taiwan

**Keywords:** *Ganoderma* triterpenoids, hyperpolarization-activated cation current, current kinetics, membrane potential, pituitary cell, heart cell

## Abstract

The triterpenoid fraction of *Ganoderma* (*Ganoderma* triterpenoids, GTs) has been increasingly demonstrated to provide effective antioxidant, neuroprotective or cardioprotective activities. However, whether GTs is capable of perturbing the transmembrane ionic currents existing in electrically excitable cells is not thoroughly investigated. In this study, an attempt was made to study whether GTs could modify hyperpolarization-activated cation currents (*I*_h_) in pituitary tumor (GH_3_) cells and in HL-1 atrial cardiomyocytes. In whole-cell current recordings, the addition of GTs produced a dose-dependent reduction in the amplitude of *I*_h_ in GH_3_ cells with an IC_50_ value of 11.7 µg/mL, in combination with a lengthening in activation time constant of the current. GTs (10 µg/mL) also caused a conceivable shift in the steady-state activation curve of *I*_h_ along the voltage axis to a more negative potential by approximately 11 mV. Subsequent addition of neither 8-cyclopentyl-1,3-dipropylxanthine nor 8-(*p*-sulfophenyl)theophylline, still in the presence of GTs, could attenuate GTs-mediated inhibition of *I*_h_. In current-clamp voltage recordings, GTs diminished the firing frequency of spontaneous action potentials in GH_3_ cells, and it also decreased the amplitude of sag potential in response to hyperpolarizing current stimuli. In murine HL-1 cardiomyocytes, the GTs addition also suppressed the amplitude of *I*_h_ effectively. In DPCPX (1 µM)-treated HL-1 cells, the inhibitory effect of GTs on *I*_h_ remained efficacious. Collectively, the inhibition of *I*_h_ caused by GTs is independent of its possible binding to adenosine receptors and it might have profound influence in electrical behaviors of different types of electrically excitable cells (e.g., pituitary and heart cells) if similar in vitro or in vivo findings occur.

## 1. Introduction

*Ganoderma* mushrooms (Língzhī in Chinese, or Reishi in Japanese) are a traditional Chinese herbal medicine that has been widely accepted as a nutritional supplement. Among many species of the mushrooms, *Gandoderm lucidum* (GL) is most commonly seen and is commercially cultivated under controlled conditions to obtain mushrooms with more consistent chemical composition [1]. GL was reported to possess a variety of biological activities for its medicinal use, such as antihypertensive, hypoglycemic and hypocholesterolemic activities among other medicinal benefits [1,2,3,4]. The primary bioactive compounds in GL were noted to include triterpenoids [4,5,6,7]. GL was also shown to prevent cardiac damage in animal models by alleviating the oxidative stress associated with myocardial injury [8].

Triterpenes are a subclass of terpenes and have a basic skeleton of C_30_. Triterpenoids have molecular weights ranging from 400 to 600 kDa, and their chemical structure is complex and noted to be highly oxidized [9]. The triterpenoid fraction of *Ganoderma*, consisting of more than 300 lanostane-tetracyclic compounds, has been growingly demonstrated to be effective at exerting an array of biological actions such as that known either to provide effective antioxidant activities for prevention of myocardial injury, or to produce neuroprotective actions [2,3,4,5,6,10,11,12,13,14,15,16,17,18,19,20,21]. *Ganoderma* triterpenoids (GTs) could also suppress inflammatory response by directly scavenging the free radicals or systemically enhancing the antioxidant enzymes, thereby lowering lipid peroxidase in chicken livers or mice [2,3,8,17,20,21,22,23]. The aqueous extract from GL was previously reported to exert anti-convulsant, anti-depressive, anxiolytic and anti-nociceptive actions [6,12,24,25]. However, whether GTs produces any perturbations on membrane ionic currents or membrane potential in electrically excitable cells (e.g., endocrine or heart cells) is not thoroughly studied.

Hyperpolarization-activated cation current (*I*_h_) has been well recognized as a key determinant of repetitive electrical activity in heart cells and in an array of sensory or central neurons, and neuroendocrine or endocrine cells [26,27,28,29,30,31,32,33,34,35]. This current is a mixed inward Na^+^/K^+^ current, which is sensitive to block by CsCl, ivabradine or zatebradine [29,36,37], and the rise in this current can consequently act to depolarize membrane potential to threshold required for the generation of action potential (AP) [28,30,31,33]. The *I*_h_ was demonstrated to be carried by channels of the hyperpolarization-activated cyclic nucleotide-gated (HCN) gene family, which belongs to the superfamily of voltage-gated K^+^ channels and cyclic nucleotide-gated channels. However, how or whether GTs is capable of interacting with HCN channels to modify the amplitude and gating of *I*_h_ is largely unknown.

Therefore, the objective of this work was to test whether GTs could exert any modifications on ionic currents (e.g., *I*_h_) present in pituitary GH_3_ cells and HL-1 cardiomyocytes. The biophysical and pharmacological properties of ionic currents, in particular *I*_h_ in these cells, were extensively characterized. Current-clamp voltage recordings were also made to evaluate whether GTs could perturb spontaneous action potentials and sag potentials present in GH_3_ cells. Findings from the present results highlight the notion that GTs can modify the amplitude and gating of *I*_h_ in a concentration-, time- and state-dependent manner.

## 2. Results and Discussion

### 2.1. Inhibitory Effect of GTs on Hyperpolarization-Activated Cation Current (I_h_) Recorded from Pituitary GH_3_ Cells

**Figure 1:**
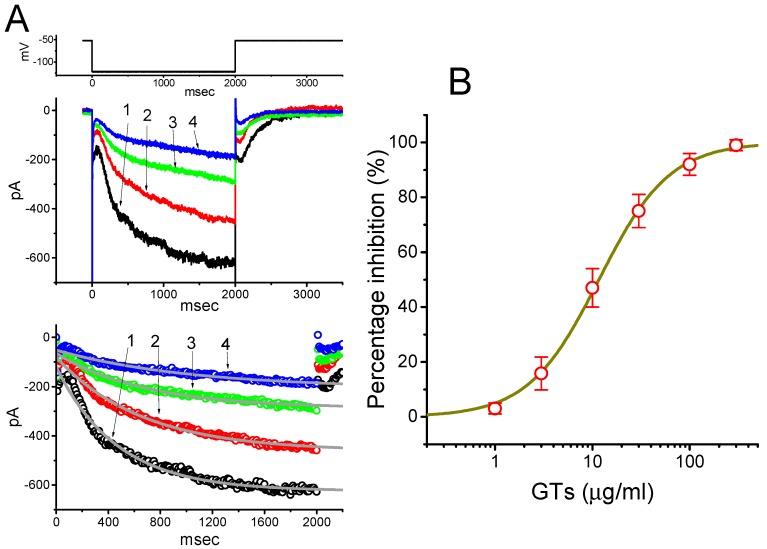
Effect of *Ganoderma* triterpenoids (GTs) on hyperpolarization-activated cation current in pituitary tumor (GH_3_) cells. In these whole-cell current recordings, cells were bathed in Ca^2+^-free, Tyrode’s solution, and we backfilled the recording electrode by using a K^+^-containing solution. The hyperpolarizing step from −52 to −122 mV was applied to the examined cells. (**A**) Superimposed *I*_h_ traces obtained in the absence (1) and presence of 10 µg/mL GTs (2), 30 µg/mL GTs (3) and 100 µg/mL GTs (4). The uppermost part is the voltage profile used. The lower part depicts the trajectory of each *I*_h_ trace shown in the upper panel (in the absence (1) and presence of 10 µg/mL GTs (2), 30 µg/mL GTs (3) and 100 µg/mL GTs (4)) was fitted by single exponential with the activation time constant (τ_act_) of 467, 657, 680 and 1037 msec, respectively. For better illustration, the data points (circles symbols) were reduced by a factor of 10. (**B**) Dose-dependent relation of GTs effect on *I*_h_ amplitude (mean ± SEM; n = 7–9 for each point). The *I*_h_ amplitudes were taken at the end of 2-sec hyperpolarizing step in the presence of different GTs doses. The continuous line was fitted with the goodness of fit by a modified Hill function described under Materials and Methods. The IC_50_ value required for GTs-induced suppression of *I*_h_ was computed to be 11.7 µg/mL.

In an initial stage of whole-cell experiments designed for the recording of *I*_h_, we bathed cells in Ca^2+^-free, Tyrode’s solution and, during the measurements, we filled the pipette by using a K^+^-containing solution. The reason why we used Ca^2+^-free, Tyrode’s solution is to eliminate contamination of Ca^2+^-activated K^+^ currents that could be possibly generated by any activity of small- or intermediate-conductance Ca^2+^-activated K^+^ channels. As shown in Figure 1A, the *I*_h_, which was identified and then characterized by the slowly activating property [27,29,38], could be readily evoked by membrane hyperpolarization to −122 mV with a duration of 2 sec. As the cells were exposed to different concentrations of GTs, the *I*_h_ amplitude was progressively decreased and the activating time course of the current also concomitantly became slowed. For example, the addition of 30 µg/mL GTs decreased *I*_h_ amplitude from 621 ± 23 to 295 ± 13 pA (*n* = 8, *p* < 0.05). In combination with these results, the value of activation time constant (τ_act_) of *I*_h_ elicited by 2-sec maintained hyperpolarization was also raised to 680 ± 25 msec (*n* = 8, *p* < 0.05) from a control value (i.e., in the absence of GTs) of 467 ± 21 msec (*n* = 8). After washout of GTs, the current amplitude was returned to 609 ± 18 pA (*n* = 7, *p* < 0.05). The dose-dependent relationship of inhibitory effect of GTs is illustrated in Figure 1B. The IC_50_ value required for GTs-mediated inhibition of *I*_h_ amplitude taken at the end of hyperpolarizing pulse was then constructed with a modified Hill equation (as detailed in Materials and Methods) and calculated to be 11.7 µM with a Hill coefficient of 1.2, and this agent at a dose of 100 µg/mL nearly abolished the *I*_h_ amplitude.

### 2.2. Effect of GTs on the Averaged I–V Relationship of I_h_ Recorded from GH_3_ Cells

**Figure 2:**
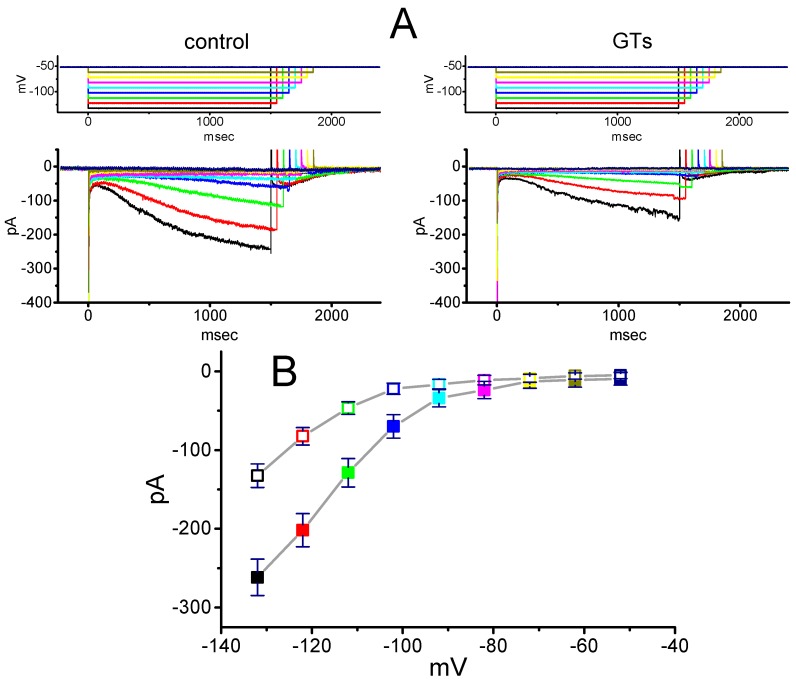
Effect of GTs on average current–voltage (*I–V*) relationship of *I*_h_ recorded from GH_3_ cells. The examined cell was maintained at −52 mV and a series of voltage steps ranging from −132 to −52 mV from a holding potential of −52 mV in 10-mV increments was thereafter applied. (**A**) Superimposed *I*_h_ traces in the absence (left, control) and presence (right) of 10 µg/mL GTs. The upper part in each panel indicates the voltage protocol used. Notably, during our voltage-clamp recordings, colored labeling in each current trace (lower part) in the absence (left) and presence (right) of GTs corresponds to that in voltage trace (upper part). (**B**) Averaged *I–V* relationships of *I*_h_ amplitude with or without the GTs addition (mean ± SEM; *n* = 8 for each point). The examined cells were voltage-clamped at −52 mV, current amplitude was then taken at the end of each hyperpolarizing step, and colored labeling in each point corresponds to that in (**A**). Closed symbols: control; Open symbols: in the presence of 10 µg/mL GTs.

We further tested whether the presence of GTs could exert any perturbations on the *I*–*V* relationship of *I*_h_ in these cells. As illustrated in Figure 2, the *I*_h_ traces were evoked by a series of voltage pulses ranging from −132 to −52 mV in 10-mV increments. Within 2 min of exposing cells to 10 µg/mL GTs, the amplitudes of *I*_h_ examined throughout the entire voltage-clamp steps (e.g., an inwardly rectifying property) were significantly decreased. For example, at the level of −132 mV, the addition of 10 µg/mL GTs reduced current amplitude from 261.5 ± 23 to 132.6 ± 14 pA (*n* = 8, *p* < 0.05). Likewise, in the presence of 10 µg/mL GTs, the whole-cell *I*_h_ conductance measured at the voltage ranging between −102 and −132 mV was decreased by 43% ± 2% to 3.67 ± 0.12 nS (*n* = 8, *p* < 0.05) from a control value of 6.48 ± 0.21 nS (*n* = 8).

### 2.3. Modification of the Steady-State Activation Curve of I_h_ Produced by the Presence of GTs

**Figure 3:**
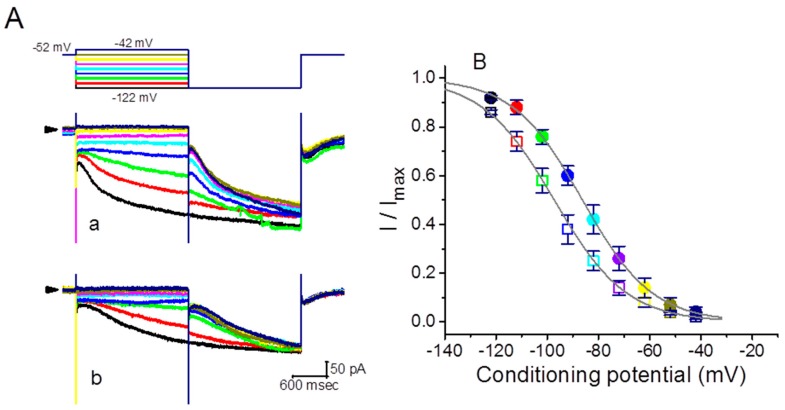
Effect of GTs on the steady-state activation curve of *I*_h_ measured from GH_3_ cells. The recording experiments were conducted in a two-step voltage pulse. The conditioning voltage pulses with a duration of 2 sec to potentials ranging from −122 and −42 mV. After each conditioning pulse, a test pulse to −122 mV with a duration of 2 sec was applied to evoke *I*_h_. (**A**) Representative *I*_h_ traces evoked by this two-step protocol. Current traces labeled a are the control, and those labeled b were obtained in the presence of 10 µg/mL GTS. The uppermost part is the voltage protocol applied, arrowhead in the left side of each panel denotes the zero current level, and calibration mark at the right lower corner applies all current traces in (**A**). Notably, colored labeling in each current trace (lower part) in the absence (a) and presence (b) of GTs corresponds to that in voltage trace (upper part). (**B**) Steady-state activation curves of *I*_h_ obtained in the absence (closed symbols) and presence (open symbols) of 10 µg/mL GTs (mean ± SEM, *n* = 9 for each point). Current amplitude was taken at the end of hyperpolarizing pulse. The sigmoidal smooth lines indicate the least-squares fit to the Boltzmann equation detailed in Materials and Methods. Colored labeling in each point corresponds to that in (**A**).

To characterize the inhibitory effect of GTs on *I*_h_ in GH_3_ cells, we further investigated its possible modifications on the steady-state activation curve of *I*_h_. Figure 3 illustrates the steady-state activation curve of *I_h_* obtained with or without addition of GTs (10 µg/mL). A two-step voltage pulse protocol created through digital-to-analog conversion was applied in this set of experiments. That is, a 2-sec conditioning pulse to various membrane potentials preceded the test pulse (2 sec in duration) to −122 mV from a holding potential of −52 mV. During the measurements, the intervals between two sets of voltage pulses were about 2 min to allow complete recovery of *I*_h_. The relationships between the conditioning potentials and the normalized amplitudes (*I*/*I*_max_) of *I*_h_ in the absence and presence of 10 µg/mL GTs were then constructed and fitted to a Boltzmann function as described under Materials and Methods. In control, *V*_1/2_ = −86.3 ± 7.1 mV, *q* = 1.81 ± 0.04 *e* (*n* = 9), whereas during cell exposure to 10 µg/mL GTs, *V*_1/2_ = −107.3 ± 6.9 mV, *q* = 1.84 ± 0.04 *e* (*n* = 9). It appeared, therefore, that the presence of GTs not only decreased the maximal conductance of *I*_h_, but also significantly shifted the activation curve along the voltage axis to a hyperpolarized potential by approximately 11 mV. However, we were unable to find significant change in the gating charge of the curve taken between the absence and presence of 10 µg/mL GTs. This occurrence indicates that the addition of GTs is capable of altering the steady-state activation curve of *I*_h_.

### 2.4. Comparisons Among the Effects of GTs, GTs Plus 8-Cyclopentyl-1,3-dipropylxanthine (DPCPX), GTs Plus 8-(p-Sulfophenyl)theophylline (8-PST), GTs Plus Oxaliplatin (OXAL) and GTs Plus Carboxiplatin (Carb) in I_h_ Amplitude

**Figure 4:**
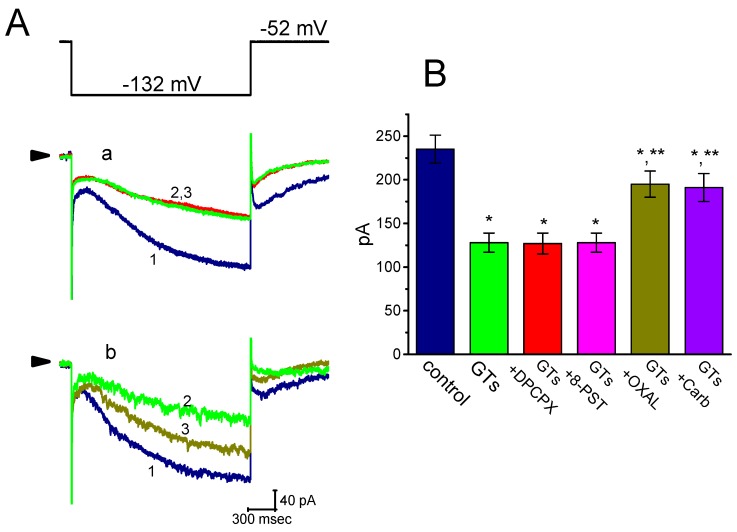
Comparisons among the effects of GTs, GTs plus 8-cyclopentyl-1,3-dipropylxanthine (DPCPX), GTs plus 8-(*p*-sulfophenyl)theophylline (8-PST), GTs plus oxaliplatin (OXAL) and GTs plus carboxiplatin (Carb) on *I*_h_ amplitude in GH_3_ cells. Current amplitude was taken at the end of 2-sec hyperpolarizing pulse to −122 mV. (**A**) Superimposed *I*_h_ traces elicited in response to step hyperpolarization (as indicated in the uppermost part). In panels a and b, current traces labeled 1 are controls and those labeled 2 were obtained in the presence of 10 µg/mL GTs alone, while in panel a or b that labeled 3 was, respectively, taken in the addition of 1 µM DPCPX (a) or 10 µM oxaliplatin (b), but still in continued presence of 10 µg/mL GTs. Arrows in current traces of each panel indicate the zero current level, and calibration mark shown in the right lower corner applies all current traces. (**B**) Summary bar graph showing the effects of GTs (10 µg/mL), GTs plus DPCPX (1 µM), GTs plus 8-PST (10 µM), GTs plus oxaliplatin (10 µM) and GTs plus carboxiplatin (10 µM) on *I*_h_ amplitude measured from GH_3_ cells (mean ± SEM; *n* = 6–9 for each bar). Current amplitude was obtained at the end of hyperpolarized pulse from −52 to −122 mV. DPCPX: 1 µM 8-cyclopentyl-1,3-dipropylxanthine; 8-PST: 10 µM 8-(*p*-sulfophenyl)theophylline; OXAL: 10 µM oxaliplatin; Carb: 10 µM carboxiplatin. *Significantly different from control (*p* < 0.05) and ^**^significantly different from 10 µg/mL alone group (*p* < 0.05).

In the next set of experiments, we further investigated whether subsequent addition of DPCPX, 8-PST, oxaliplatin or carboxiplatin, but still in continued presence of GTs, was able to modify GTs-mediated inhibition of *I*_h_ inherently in GH_3_ cells. As shown in Figure 4, further application of neither DPCPX (1 µM) nor 8-PST (10 µM) effectively perturbed the inhibition of *I*_h_ produced by 10 µg/mL GTs, despite the ability of GTs to suppress *I*_h_ amplitude together with the slowing in current activation. DPCPX or 8-PST was reported to be antagonist of adenosine A_1_ receptors [39]. However, in continued presence of 10 µg/mL GTs, further addition of oxaliplatin (10 µM) or carboxiplatin (10 µM) was able to reverse GTs-mediated effectiveness in the suppression of *I*_h_. Oxaliplatin was recently reported to activate HCN-encoded current [40].

### 2.5. Effects of GTs on the Firing of Spontaneous Action Potentials (APs) Recorded from GH_3_ Cells

**Figure 5:**
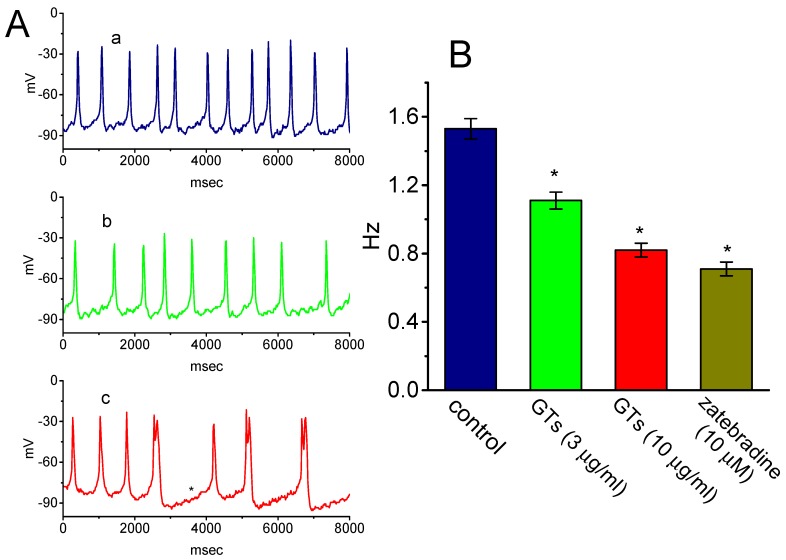
Effect of GTs on the firing of spontaneous action potentials (APs) in GH_3_ cells. Cells were bathed in normal Tyrode’s solution containing 1.8 mM CaCl_2_, the pipette used was filled with K^+^-containing solution, and current-clamp voltage recordings were made. (**A**) Potential trace obtained in the absence (a) and presence of 3 µg/mL GTs (b) or 10 µg/mL GTs (c). The asterisk in potential trace labeled c indicates the depressed pacemaker potential. (**B**) Summary bar graph showing the effects of GTs and zatebradine (10 µM) on the firing frequency of spontaneous APs (mean ± SEM; *n* = 8 for each bar). *Significantly different from control (*p* < 0.05).

In another set of experiments, we further tested whether GTs produces any effectiveness in spontaneous APs inherently in GH_3_ cells [31,38,41]. Cells were immersed in normal Tyrode’s solution containing 1.8 mM CaCl_2_ and current-clamp voltage recordings were performed to measure the occurrence of spontaneous APs. Of note, as cells were exposed to GTs, the firing of spontaneous APs was progressively decreased (Figure 5). Within 2 min of exposing cells to 10 µg/mL GTs, the resting potential was shifted to the hyperpolarized direction, in combination with the decreased frequency of spontaneous APs and with appearance of depressed pacemaker potential. Similarly, the addition of zatebradine (10 µM), an inhibitor of *I*_h_ [36,42], was also found to depress the firing frequency of APs significantly. Therefore, changes in the frequency of spontaneous APs produced by either GTs or zatebradine could conceivably be linked to a mechanism via the inhibition of *I*_h_ described above.

### 2.6. Effect of GTs on Sag Potential in GH_3_ Cells

**Figure 6:**
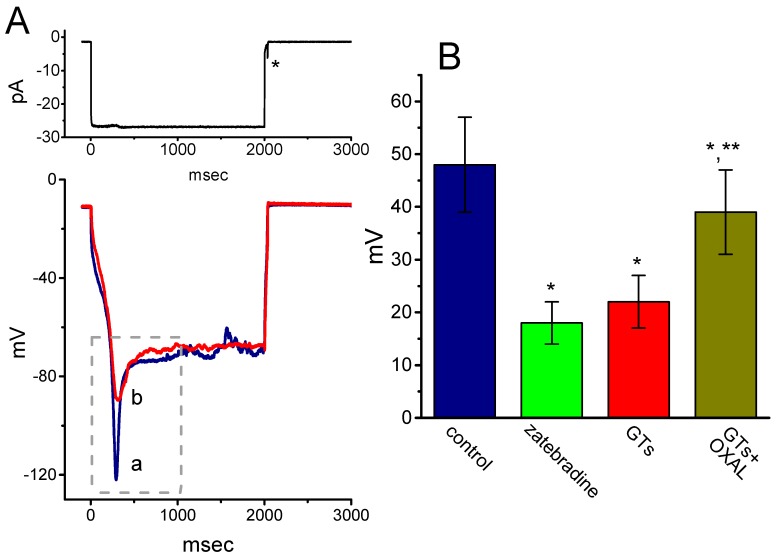
Effect of GTs on sag potential recorded under the current-clamp configuration in GH_3_ cells. Cells were bathed in normal Tyrode’s solution and we filled the recording pipette with K^+^-containing solution. As the whole-cell recordings were established, the experiment was swiftly switched to current-clamp condition with 2-sec hyperpolarizing current stimuli. (**A**) Potential traces obtained in the absence (a) and presence (b) of 10 µg/mL GTs. Dashed box indicates the sag potential in response to long-lasting hyperpolarizing current (as indicated in the upper part), and the asterisk is the action current taken as membrane potential return to the resting potential. (**B**) Summary bar graph showing the effects of zatebradine (10 µM), GTs (10 µg/mL) and GTs (10 µg/mL) plus oxaliplatin (OXAL, 10 µM) on the amplitude of sag potential in GH_3_ cells (mean ± SEM; *n* = 7 for each bar). *Significantly different from control (*p* < 0.05) and **significantly different from GTs alone group (*p* < 0.05).

Another set of current-clamp recordings was next made in attempts to evaluate the possible presence of sag potential, which is closely linked to the emergence of *I*_h_ [43]. As shown in Figure 6, under our experimental condition, when the whole-cell voltage recordings were firmly established, hyperpolarizing current injection with the amplitude of around 25 pA was found to induce sag potential (i.e., drop down to a lower level in the membrane potential upon hyperpolarizing current stimuli). The addition of zatebradine or GTs was effective at decreasing the amplitude of sag potential, while chlorotoxin (1 µM) was unable to modify sag potential in these cells. For example, cell exposure to GTs (10 µg/mL) decreased the amplitude from 48 ± 9 to 22 ± 5 mV (*n* = 7, *p* < 0.05). Moreover, subsequent application of oxaliplatin (10 µM), still in the presence of GTs (10 µg/mL), attenuated GTs-induced suppression of sag potential, as evidenced by a significant increase of sag potential to 39 ± 8 mV (*n* = 7, *p* < 0.05). However, chlorotoxin (1 µM) alone, a blocker of Cl^−^ channels, did not produce any effect on sag potential in these cells (data not shown). Therefore, the depression of sag potential caused by GTs could result largely from its inhibitory effect on *I*_h_ observed in GH_3_ cells.

### 2.7. Effect of GTs on I_h_ Recorded from HL-1 Cardiomyocytes

**Figure 7:**
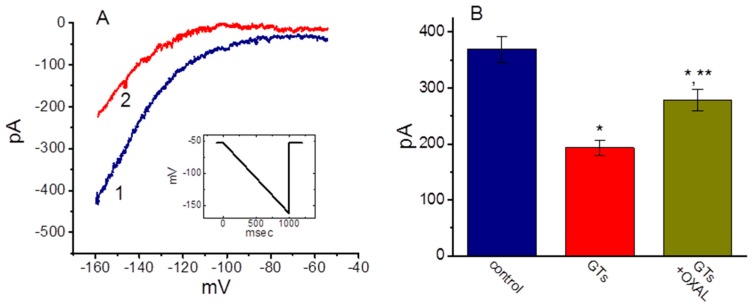
Effect of GTs on *I*_h_ elicited by a downsloping ramp pulse in HL-1 cardiomyocytes. In these experiments, the examined cell was maintained at −52 mV and the linear ramp pulse from −52 to −162 mV with a duration of 1 sec (as indicated in inset of (**A**)). (**A**) Representative *I*_h_ traces in response to such linear ramp obtained with or without the addition of GTs. Current trace labeled 1 is control, and that labeled 2 was obtained 2 min after addition of 10 µg/mL GTs. (**B**) Summary bar graph showing effect of GTs and GTs plus oxaliplatin on *I*_h_ elicited by ramp pulse. Current amplitude was measured at the level of −152 mV. Each bar indicates the mean ± SEM (*n* = 7–8). * Significantly different control (*p* < 0.05) and ^**^ significantly different from 10 µg/mL GTs alone group (*p* < 0.05).

It has been shown that GL might prevent cardiac damage by decreasing the oxidative stress associated with myocardial injury [8]. We therefore also tested whether GTs exerts any effects on *I*_h_ present in HL-1 heart cells [44,45]. These experiments were conducted in cells bathed in Ca^2+^-free Tyrode’s solution, and the downsloping ramp pulse from −52 to −162 mV with a duration of 1 sec was applied to evoke *I*_h_ in these cells. As shown in Figure 7, the *I*_h_ evoked by such long-lasting ramp pulse was subject to be suppressed by the addition of GTs. For example, as the cells were exposed to 10 µg/mL GTs, current amplitude measured at the level of −152 mV was profoundly decreased to 193 ± 14 pA (*n* = 8, *p* < 0.05) from a control level of 369 ± 23 pA (*n* = 8). Likewise, under such a downsloping ramp pulse, the presence of 10 µg/mL GTs profoundly decreased the whole-cell *I*_h_ conductance measured ranging between −152 and −132 mV from 7.72 ± 0.16 to 4.2 ± 0.12 nS (*n* = 8, *p* < 0.05). Moreover, the addition of 10 µM oxaliplatin, still in the presence of 10 µg/mL GTs, attenuated current amplitude suppressed by GTs, as evidenced by a significant increase in *I*_h_ amplitude to 278 ± 19 pS (*n* = 8, *p* < 0.05). In DPCPX-treated HL-1 cells, GTs-mediated inhibition of *I*_h_ remained unchanged (data not shown). Therefore, consistent with the observations made above in GH_3_ cells, the GTs addition effectively inhibited the amplitude of *I*_h_ in response to long-lasting membrane hyperpolarization in HL-1 cardiomyocytes.

The present results demonstrated that the inhibition by GTs of *I*_h_ in GH_3_ cells did not simply decrease current magnitude but also altered the kinetics of the current, thereby indicating that it is able to produce a dose-, time- and state-dependent activation of *I*_h_. The steady-state activation curve of *I*_h_ in the presence of GTs was shifted along voltage axis toward the less depolarized potential. The IC_50_ value (i.e., 11.7 µg/mL) required for GTs-mediated inhibition of *I*_h_ observed in GH_3_ cells is similar to that used for anti-oxidative or neuroprotective properties [2,3,4,15,16,17,46,47]. Such intriguing actions could be of pharmacological relevance and appear to be upstream of its effects on oxidative stress occurring inside the cell.

Previous works have demonstrated that GTs could abundantly contain various nucleosides including adenosine [48,49,50]. Adenosine has been previously reported to modify the amplitude of *I*_h_ in heart cells (i.e., sinoatrial cells) [26,51]. However, further application of neither DPCPX nor 8-PST, still in the presence of GTs, was capable of attenuating GTs-mediated inhibition of *I*_h_ effectively. DPCPX or 8-PST was previously reported to be blocker of adenosine A_1_ receptor [39]. Moreover, subsequent addition of oxaliplatin or carboxiplatin, in continued presence of GTs, significantly reversed the *I*_h_ amplitude suppressed by GTs. Oxaliplatin was recently found to activate HCN-encoded current [40]. Subsequent addition of adenosine deaminase (2 unit/mL), in continued presence of GTs, did not modify GTs-mediated inhibition of *I*_h_ in GH_3_ cells (data not shown). Consequently, it seems unlikely that GTs-mediated block of *I*_h_ observed in GH_3_ cells ascribes predominantly from nucleosides (e.g., adenosine) possibly contained in the ingredients of GTs. 

Previous studies have demonstrated the ability of GTs to have an inhibitory effect on the activity of acetylcholinesterase [4,47]. However, we were unable to find that further application of atropine (10 µM), an antagonist of muscarinic receptors, could modify GTs-mediated suppression of *I*_h_ in GH_3_ or HL-1 cells (data not shown). It seems unlikely that such inhibition could be connected with the suppression of acetylcholinesterase activity. Alternatively, a previous report showed the ability of the active ingredients in GL to modify the cAMP signaling [52]. However, GTs-mediated inhibition of *I*_h_ in GH_3_ cells could not be reversed by further addition of SQ-22536 (10 µM), an inhibitor of adenylate cyclase, (data not shown), suggesting that such an inhibitory action is apparently unlinked to the GTs effectiveness in cAMP signaling.

There is growing evidence to show that inflammatory pain could be closely linked to the magnitude of *I*_h_ expressed in peripheral sensory neurons [40]. GTs was also previously reported to exert analgesic actions [11,53], which could be possibly associated with its inhibition of *I*_h_ in different types of sensory neurons. It is also important to note that the *I*_h_ was expressed either in urinary bladder, ureter and renal pacemaker tissue or in different types of tumor cells (e.g., lung carcinoma cells) [34,54,55,56,57]; at extent GTs-induced block of *I*_h_ is linked to its antineoplastic actions or impairments in urine excretion remains to be imperatively evaluated.

In addition to the reduction of *I*_h_ amplitude, the τ_act_ value for *I*_h_ activation observed in GH_3_ cells was virtually raised in the presence of GTs. Although the detailed mechanism of GTs actions on *I*_h_ is largely unresolved, these triterpenoids are likely to have greater affinity to the open/inactivated state than to the resting state residing in HCN channels, thus leading to a clear modification in the amplitude and gating of *I*_h_. Nonetheless, GTs-mediated inhibition of *I*_h_ can account largely for its suppression in the firing of spontaneous APs as well as in the amplitude of sag potential induced by long-lasting hyperpolarizing current stimuli.

There are four mammalian subtypes (HCN1, HCN2, HCN3 and HCN4), which have been cloned to date [30,33,58]. It is possible that HCN2, HCN3 or mixed HCN2 + HCN3 channels are functionally expressed in GH_3_ or other types of endocrine cells [31,33]. Due to the importance of *I*_h_ (i.e., KCNx-encoded currents) in contributing to excitability and automaticity of electrically excitable cells [27,28,29,31,41], findings from this study could provide novel insights into electrophysiological and pharmacological properties of GTs and other structurally related triterpenoids. Indeed, under our current-clamp recordings, addition of GTs was found to reduce the firing frequency of APs as well as sag potentials recorded from GH_3_ cells. The reduction of AP firing caused by GTs could be primarily explained by its clear modifications in the amplitude and gating of *I*_h_. However, it is important to note that the inhibitory effects of GTs on *I*_h_ seen in GH_3_ or HL-1 cells tend to be not isoform-specific. To what extent GTs-mediated change in endocrine cells [59] is linked to its suppression of *I*_h_ observed in GH_3_ cells remains to be determined. Moreover, whether GTs-induced bradycardia or cardioprotective effect [11,13,15] is intimately linked to its inhibitory effect on the amplitude and gating of *I*_h_ in heart cells also imperatively needs to be studied.

The present results demonstrated that in HL-1 atrial cardiomyocytes, the addition of GTs was efficacious at suppressing *I*_h_ elicited by a downsloping ramp pulse. A leftward shift in the steady-state activation curve of *I*_h_ in GH_3_ cells was demonstrated in its presence. Notably, recent reports have shown the ability of ivabradine, an inhibitor of *I*_h_, to exert beneficial effects on post-resuscitation myocardial dysfunction [11,13,15,18,60]. It is thus tempting to anticipate that GTs-mediated inhibition of *I*_h_ in heart cells could be closely linked to its beneficial effect on cardiac function.

## 3. Materials and Methods

### 3.1. Chemicals, Drugs and Solutions

Adenosine deaminase, atropine, oxaliplatin and 8-(*p*-sulfophenyl)theophylline (8-PST) were obtained from Sigma-Aldrich (St. Louis, MO, USA), and 8-cyclopentyl-1,3-dipropylxanthine (DPCPX), SQ-22536, and zatebradine were from Tocris Cookson Ltd. (Bristol, UK), and carboxiplatin was from Pfizer (New Taipei City, Taiwan). *Ganoderma* triterpenoids (GTs) was kindly provided by Dr. Fan-E Mo, Department of Cell Biology and Anatomy, College of Medicine, National Cheng Kung University, Tainan, Taiwan, while chlorotoxin was by Dr. Woei-Jer Chuang, Department of Biochemistry, College of Medicine, National Chung Kung University, Tainan, Taiwan). GTs was dissolved in dimethyl sulfoxide (less than 0.01%) and made immediately prior to experiments. Unless stated otherwise, tissue culture media, horse serum, fetal bovine or calf serum, l-glutamine and trypsin/EDTA were acquired from Invitrogen (Carlsbad, CA, USA), while all other chemicals, such as CsCl, HEPES and aspartic acid, were of the best available quality, mostly at analytical grades.

The normal Tyrode’s solution used in this study contained (in mM): NaCl 136.5, KCl 5.4, CaCl_2_ 1.8, MgCl_2_, 0.53, glucose 5.5 and HEPES-NaOH buffer 5.5 (pH 7.4). In whole-cell clamp experiments of recording membrane potential or *I*_h_, we backfilled the pipette by using a solution (in mM): K-aspartate 130, KCl 20, KH_2_PO_4_ 1, MgCl_2_, EGTA 0.1, Na_2_ATP 3, Na_2_GTP 0.1 and HEPES-KOH buffer 5 (pH 7.2). The pipette solution and culture medium were commonly filtered on the day of use with Acrodisc^®^ syringe filter with 0.2 µm Supor^®^ membrane (Pall Corp., Port Washington, NY, USA).

### 3.2. Cell Preparations

Pituitary tumor GH_3_ cells, obtained from the Bioresources Collection and Research Center ((BCRC-60015); Hsinchu, Taiwan), were maintained in Ham’s F-12 medium supplemented with 15% horse serum, 2.5% fetal calf serum and 2 mM l-glutamine, while the HL-1 atrial cell line was derived from the AT-1 mouse atrial cardiomyocyte tumor lineage, which was originally obtained from Louisiana State University in New Orleans, LA, USA. These cells were known to retain differentiated phenotypes of adult atrial myocytes [44,45]. Cells were maintained in Claycomb medium (Sigma-Aldrich) supplemented with 10% fetal bovine serum, 100 U/mL penicillin, 100 mg/mL streptomycin, 100 µM norepinephrine and 2 mM l-glutamine (Chang and Wu, 2018). GH_3_ or HL-1 cells were grown in a humidified environment of 5% CO_2_/95% air. In a separate set of experiments, HL-1 cells were treated with DPCPX (1 µM) at 37 °C for 6 h.

### 3.3. Electrophysiological Measurements

Before the experiments, cells (i.e., GH_3_ or HL-1 cells) were gently dispersed and a few drops of cell suspension were shortly transferred to a home-made chamber mounted on the fixed stage of inverted Diaphot-200 microscope (Nikon, Tokyo, Japan). They were immersed at room temperature (20–25 °C) in normal Tyrode’s solution, the composition of which is described above. We fabricated the recording pipette from Kimax-51 capillary tubes (#34500; Kimble, Vineland, NJ, USA) using a vertical PP-83 puller (Narishige, Tokyo, Japan), and their tips were then fire-polished with MF-83 microforge (Narishige, Tokyo, Japan). During the measurements, the electrode with tip resistance of 3–5 MΩ, which was firmly inserted into holder, was maneuvered by use of WR-98 micromanipulator (Narishige, Tokyo, Japan). Patch-clamp experiments were measured in whole-cell arrangement (i.e., voltage- or current-clamp condition) by using an RK-400 patch-clamp amplifier (Bio-Logic, Claix, France) connected with an HP Pavilion X360 touchscreen laptop computer (14-cd1053TX; Hewlett-Packard, Palo Alto, CA, USA) [39,61]. Shortly before giga-seal formation was achieved, the potentials were corrected for the liquid junction potential that developed at the pipette tip, as the composition of pipette solution was different from that in the bath. The whole-cell results were corrected by the liquid junction potential measured under our experimental conditions (−12.5 ± 0.3 mV, *n* = 14).

The dose-dependent relation of GTs on the inhibition of *I*_h_ was least-squares fitted to the Hill equation by using nonlinear regression analysis (64-bit OriginPro 2016; Microcal, Northampton, MA). That is,

$$\mathrm{Percentage}\;\mathrm{inhibition}\;=\;\left(E_{max}\;\times \;GTs^{n_{H}}\right)/\left(IC_{50}^{n_{H}}+GTs^{n_{H}}\right),$$

where GTs represents the dose (µg/mL) of GTs; IC_50_ and n_H_ are the concentrations required for a 50% inhibition and the Hill coefficient, respectively and *E*_max_ is GTs-induced maximal suppression of *I*_h_.

To characterize the inhibitory action of GTs on *I*_h_, the steady state activation curve of the current was constructed using a two-step protocol. The relationships between the conditioning potentials and the normalized amplitudes of *I*_h_ with or without the addition of 10 µg/mL GTs were fitted with the goodness of fit by a Boltzmann function in the following form:

$$\frac{I}{I_{max}}\;=\;\frac{1}{1+exp\left[\frac{\left(V-V_{\frac{1}{2}}\right)qF}{RT}\right]},$$

where *I*_max_ is the maximal activated *I*_h_; *V* is the conditioning potential in mV; *V*_1/2_ is the membrane potential (in mV) for half-maximal activation; *q* is the apparent gating charge and *F*, *R* or *T* is Faraday’s constant, the universal gas constant or the absolute temperature, respectively.

### 3.4. Statistical Analyses

To perform linear or non-linear (e.g., sigmoidal or exponential function) curve fitting to the data set (i.e., the goodness of fit) was implemented by using either OriginPro 2016 (OriginLab, Northampton, MA, USA), pCLAMP 10.7 (Molecular Devices, San Jose, CA, USA) or Prism version 5.0 (GraphPad, La Jolla, CA, USA). Data were analyzed and are plotted using OriginPro (OriginLab), and they were expressed as mean ± standard error of the mean (SEM). The paired or unpaired Student’s *t*-test, or one-way analysis of variance (ANOVA) followed by post-hoc Fisher’s least-significance difference test for multiple comparisons, were implemented for the statistical evaluation of differences among means. We further used the non-parametric Kruskal–Wallis test, when the assumption of normality underlying ANOVA could be violated. Statistical analyses were performed by using IBM SPSS^®^ version 20.0 (IBM Corp., Armonk, NY, USA). *p* < 0.05 was considered significant.

## 4. Conclusions

Although additional studies are needed to further validate our experimental results on different other types of cells, GTs-mediated inhibition of *I*_h_ shown here could be an unidentified but the important ionic mechanism underlying the depressed excitability of GH_3_ cells or HL-1 cardiomyocytes. However, these appreciable actions on *I*_h_ presented herein are not explained solely by its possible binding to adenosine receptors.

## Abbreviations

AP	action potential
DPCPX	8-cyclopentyl-1,3-dipropylxanthine
GL	*Gandoderm lucidum* (Língzhī or Reishi)
GTs	*Ganoderma* triterpenoids
HCN gene	hyperpolarization-activated cyclic nucleotide-gated gene
*I-V*	current versus voltage
IC_50_	the concentration required for half-maximal inhibition
*I*_h_	hyperpolarization-activated cation current
8-PST	8-(*p*-sulfophenyl)theophylline
τ_act_	activation time constant
